# Successful Use of Continuous Veno-Venous Haemodialysis in a Case of Potential Lethal Caffeine Intoxication

**DOI:** 10.3390/toxics11020196

**Published:** 2023-02-20

**Authors:** Elles J. Reimerink, Daan W. Huntjens, Lindsey G. Pelkmans, Jan-Willem H. J. Geerts, Eric J. F. Franssen

**Affiliations:** 1 Department of Intensive Care, OLVG Hospital, 1061 AE Amsterdam, The Netherlands; 2 Department of Clinical Pharmacy, OLVG Hospital, 1061 AE Amsterdam, The Netherlands

**Keywords:** caffeine intoxication, continuous veno-venous haemodialysis, caffeine clearance

## Abstract

Here we describe the case of a potentially lethal caffeine intoxication after the reported ingestion of 10 g of caffeine. Due to hemodynamic instability with tachycardia and hypertension with an insufficient effect of continuous labetalol infusion, the patient was started on continuous veno-venous haemodialysis (CVVHD). After successful treatment for 15 h, CVVHD could be discontinued, and the patient was discharged home the next day. This case report is the first to report the use of CVVHD as a haemodialysis modality in the case of caffeine intoxication and illustrate the effect on caffeine clearance. We stress the importance of an early recognition of caffeine intoxication, so that haemodialysis can be considered in the case of a potentially lethal intoxication.

## 1. Introduction

Caffeine (1,3,7-trimethylxanthine, guaranine) is a plant-derived alkaloid that antagonizes subtype A1 and A2a of the adenosine receptor. Since adenosine is an endogenous neuromodulator with mostly inhibitory effects, the antagonism of adenosine results in positive inotropic and chronotropic effects. After oral intake, caffeine is completely absorbed in the stomach and intestines (100%) and is almost completely metabolized in the liver by CYP1A2 (95%) through demethylation of caffeine (1,3,7-trimethylxanthine) to paraxanthine (1,7-dimethylxanthine) [1]. In the case of regular consumption, the metabolization will follow linear kinetics with a half-life time of 3-5 h and a plasma protein binding of 17–36% [2]. However, due to saturation of CYP1A2 in the case of an overdose, the unbound fraction will no longer be dose-linear, and caffeine will follow non-linear kinetics, with a higher unbound fraction and a prolonged half-life time [3,4]. After metabolization, the majority of caffeine is eliminated in the urine through renal excretion (85–88%) [4].

Caffeine is usually consumed to increase focus, enhance memory, and improve physical performance [2,5,6]. Caffeinated beverages contain varying amounts of caffeine: from 60 mg in a cup of coffee to 250 mg in potent energy drinks. The consumption of these beverages rarely leads to severe caffeine intoxication. However, caffeine supplements regularly exceed this dosage and can contain 300 mg each. These supplements can be bought limitlessly online as pills or as pure caffeine powder. The increasing popularity of caffeine supplements has resulted in (unintended) severe caffeine intoxications, which have necessitated treatment in a critical care unit and even resulted in deaths [7,8]. The American Association of Poison Control Centers (AAPCC) reported 3031 cases of single-substance caffeine use in 2019, of whom 415 were treated in a healthcare facility, resulting in 23 major outcomes (life-threatening or resulting in significant residual disability or disfigurement) [9].

In previous case reports, the ingestion of 15–30 mg/Kg has resulted in significant toxicity, and oral doses of >5 g have been reported as fatal [1,6]. Due to the severity of the intoxication, elimination enhancement through renal replacement therapy (RRT) can prove a valuable therapy. Since caffeine is a small hydrophilic molecule with a low plasma protein binding (17–36%) and a small volume of distribution of 0.61 L/Kg, it is suitable for dialysis [10]. Successful intermittent haemodialysis has been described in case reports; however, the benefit of continuous renal replacement therapy remains unclear.

We here describe a case of severe caffeine intoxication with a potentially lethal oral dosage of 10 g, in which the patient has made a full recovery after treatment with continuous veno-venous haemodialysis (CVVHD). To the best of our knowledge, this is the first case report describing the use of CVVHD as a haemodialysis modality in caffeine intoxication.

## 2. Case Report

A 29-year-old male patient with a history of borderline personality disorder and alcohol and drug abuse presented to the cardiac care unit (CCU) with complaints of chest pain and stomach ache. As an experiment, he voluntarily consumed 10 g of caffeine powder approximately 14 h before presentation. About 30 min after ingestion, he started vomiting and sweating, and he experienced a heavy chest. These symptoms endured 13 h until he eventually called the emergency services and was taken to the hospital. At presentation, the patient was anxious and in pain, and he was sweating profoundly. Vital signs showed tachycardia (152 beats per minute), hypertension (190/35 mmHg), tachypnoea (22 breaths per minute), an oxygen saturation of 97%, and a subfebrile temperature (38 °C). The electrocardiogram showed sinus tachycardia (148 beats per minute) with a prolonged QTc (554 ms) and ST-depressions in II, V2–V5 and ST-elevation in Avr, with normal R-wave progression. The initial laboratory results are depicted in Table 1.

**Figure 1:**
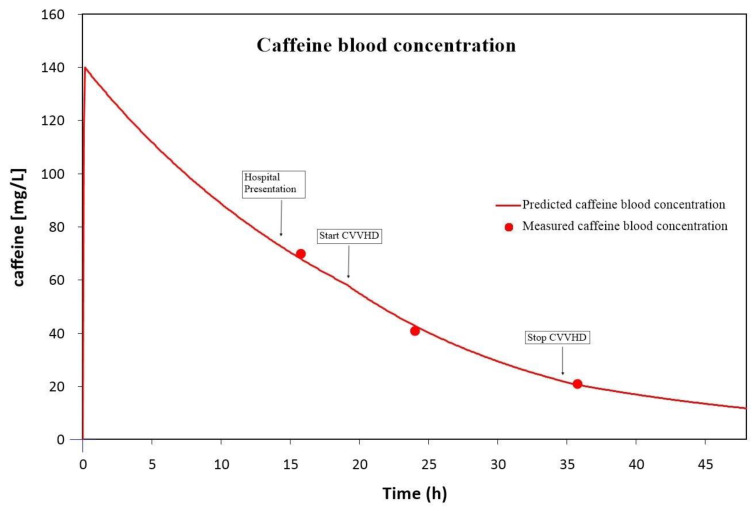
Caffeine blood concentration based on three caffeine blood samples extrapolated in a pharmacokinetic model using a therapeutic drug-management application.

Despite the presence of lactate acidosis, arterial blood gas analysis showed metabolic alkalemia due to continuous vomiting. The drugs-of-abuse toxicology screening in a urine sample (Quidel, Tox Drug Screen, 94600) was found positive for benzodiazepines and cocaine. The positive benzodiazepine screening was attributable to midazolam administered in the ambulance. Cocaine and benzoylecgonine (a metabolite of cocaine) were not detectable in serum via liquid chromatography coupled with multistage accurate mass spectrometry (LC-MS^n^) and were therefore not clinically relevant. Initial treatment included infusion of isotonic liquids, electrolyte supplementation, and anti-emetics (including dexamethasone 12 mg). Despite supportive therapy, tachypnoea increased (40 breaths per minute), and the temperature rose to a fever (38.7 °C), in addition to persisting nausea, tachycardia and hypertension. Subsequently, the patient was admitted to the intensive care unit, where benzodiazepines and a continuous infusion of labetalol were initiated to treat anxiety and excesses in haemodynamics, respectively. In addition, CVVHD was initiated to increase caffeine elimination, using the MultifiltratePRO (Fresenius medical care) with an ultraflux AV1000S filter and regional citrate anticoagulation. The dialysate flow rate was set at 2 L/h and the blood flow rate at 100 mL/min at a temperature of 37 °C. Within three hours after initiating CVVHD, the fever resolved due to active cooling. Tachypnoea started declining some hours later. The follow-up of elevated cardiac blood markers showed a decrease six hours later. After 15 h of CVVHD, the patient’s haemodynamics normalized. Therefore, labetalol could be withdrawn, and CVVHD was discontinued. Caffeine blood levels were taken at presentation, during CVVHD treatment and after completion of therapy, and are depicted in Figure 1. The caffeine serum concentration upon presentation was 70 mg/L and had dropped to 21 mg/L after CVVHD treatment. Using the therapeutic drug-management application MwPharm version 1.8.2.20 (Mediware), we were able to simulate the serum caffeine concentrations (see Figure 1 and Table 2) [11]. After 30 h of ICU admission, the patient was discharged to the internal medicine ward with telemetric monitoring of the heart rhythm and discharged home the next day.

## 3. Discussion

Despite the increasing popularity of energy drinks, most deaths resulting from caffeine overdose can be attributed to the consumption of diet pills and stimulants [12]. Initial symptoms of intoxication include nausea, vomiting, tachycardia, hypertension, hypotension, and convulsions. Secondary hypokalaemia, hyperglycaemia, hypocalcaemia, lactate acidosis, and rhabdomyolysis with subsequent kidney failure have been described. These biochemical changes and an increased sympathetic activity can result in cardiovascular complications, including potentially lethal arrhythmia and myocardial infarction [2,13,14]. Therapy includes preventing systemic absorption through gastric lavage and active charcoal combined with a laxative and should be initiated within 30–90 min after intake, based on caffeine’s half-life time. However, since the half-life time can be prolonged in case of an overdose, active charcoal is possibly effective even after this period. Furthermore, therapy focuses on supportive care, such as electrolyte correction, fluid resuscitation, anti-emetics, beta-blockade and benzodiazepines, in the case of moderate to severe anxiety or convulsions [15]. These therapies require continuous monitoring of vital signs and biochemical markers. Therefore, admittance to a critical care unit is highly recommended in case of severe caffeine intoxication.

There are limited publications about using RRT in caffeine intoxication. Theophylline is also a xanthine derivate. It shows, structurally and in pharmacology, many similarities to caffeine. There are more publications about the use of RRT in theophylline intoxication. The EXtracorporeal TReatments In Poisoning workgroup (EXTRIP) published a guideline and recommended extracorporeal treatment in severe theophylline poisoning [16]. Clinical conditions can classify this severity (e.g., seizures or shock), as can a plasma concentration >100 mg/L. Intermittent haemodialysis is the preferred and recommended extracorporeal treatment, but hemoperfusion or CRRT (continuous renal replacement therapy), including CVVHD, are acceptable alternatives if haemodialysis is not available. In the article by Kim et al. [17], the authors provide a clear view on the differences between CRRT and haemodialysis in patients with an intoxication. The different extra-corporeal treatments are compared in the case of several well-known drug intoxications. The authors conclude that haemodialysis is the more effective treatment with all intoxications. The Dutch guidelines for intoxications agree with these conclusions but name CRRT as an acceptable suboptimal alternative [10]. The exceptions to this conclusion are patients who are haemodynamically unstable and in situations where haemodialysis is not available in the acute setting. Another indication for starting CRRT could be after the initial haemodialysis session to prevent a rebound rise of the drug. Although CRRT is less effective than haemodialysis, CRRT can be considered in intoxications with drugs that have a low protein binding, low volume of distribution (VD) and narrow therapeutic index. Examples of drugs where CRRT can be an acceptable alternative in intoxications are lithium, methanol and ethylene glycol.

The severity of intoxication, in this case, was based on the reported ingested amount and clinical presentation. Since death due to severe caffeine intoxication has been described after an oral intake of 5 g or more of caffeine [1,6], this patient’s consumption of 10 g of caffeine was potentially lethal. Similarly, the predicted initial serum concentration was 140 mg/L, where concentrations of >80-100 mg/L are potentially lethal [2,8]. Based on the high ingested amount of caffeine, the high predicted blood levels, and the severity of the symptoms presented by our patient, we initiated CVVHD. Criteria for initiating haemodialysis in case of caffeine intoxication have been described by others [18]. The authors suggest that patients with severe clinical symptoms, in our case a tachycardia of >140 beats per minute, and high caffeine concentrations ≥140 mg/L can benefit from haemodialysis. Due to a delay in presentation, however, the initial serum concentration turned out to be 70 mg/L and did not therefore meet the suggested criteria. Even so, a concentration of 70 mg/L must still be considered a severe intoxication, since toxicity is seen at a caffeine serum concentration of >50 mg/L. The elevated initial serum concentration, even after a delay in presentation, illustrates the slow clearance of caffeine in case of an overdose. High serum caffeine concentrations result in the saturation of CYP1A2. Consequently, caffeine clearance follows non-linear kinetics in case of an overdose, thereby prolonging the half-life time from 5 h to >16 h, and up to 120 h has been described [2,3]. In this case, the half-life time was prolonged to 15 h (Figure 1), three times the half-life time of regular consumption. Due to this long half-life time, in combination with low VD and low plasma protein binding, toxin removal through CVVHD can be a valuable intervention to increase clearance and improve symptoms.

Caffeine concentrations can be quantified using immunoassays (ELISA) or high-performance liquid chromatography (HPLC). Even though ELISA may be more cost-effective, HPLC is the method of choice, since it has a high sensitivity and specificity [19,20]. In case of an intoxication, a high specificity is requested, since interference with other xanthine derivatives should be avoided. Sensitivity is less relevant, since intoxications generally concern high drug levels, which are measured to estimate the duration to a return to therapeutic drug levels and an indication for dialysis. Following the validated sensitivity and the high specificity of HPLC, we recommend HPLC as the quantification method of choice when used in the case of caffeine intoxication.

The effect of CVVHD is graphically illustrated in Figure 1*,* where the deflection in the curve at t = 19 h shows the additional effect of CVVHD in addition to the patient’s clearance capacity. A more pronounced result can be expected using intermittent haemodialysis compared to CVVHD [3,21]. However, emergency haemodialysis is not always logistically possible or can be contra-indicated because of haemodynamic instability. We have shown that CVVHD can be a helpful alternative. Using high blood flow rates and high dialysis flow rates theoretically maximizes the effect of CVVHD on caffeine clearance; however, further studies are needed to test this hypothesis. Additionally, the effect of CVVHD can be more pronounced in the case of higher caffeine serum concentrations. Given caffeine’s low plasma protein binding (17–36%), a higher plasma concentration will most likely lead to a higher percentage of unbound caffeine due to protein saturation. Since unbound caffeine is dialyzable, higher caffeine concentrations may result in additional serum clearance. Intermittent haemodialysis or CVVHD should therefore be reserved for cases involving severe, potentially lethal caffeine toxicity, hence with a high percentage of unbound caffeine. Additionally, a fast initiation of therapy will add to the effectiveness of CVVHD and therefore to its effectiveness in relation to symptoms of caffeine toxicity. We therefore stress the importance of an early recognition of caffeine toxicity. Once initiated, the duration of CVVHD therapy can best be adjusted to clinical response.

## 4. Conclusions

CVVHD may be an acceptable alternative to intermittent haemodialysis in the case of severe, potentially lethal caffeine intoxication, when intermittent haemodialysis is not available. Preferred treatment should always be haemodialysis, if possible. Overall CVVHD can be considered in intoxications with drugs that have low protein binding, low VD and a narrow therapeutic index. Adding Cytosorb could further improve effectivity. Both modalities should be reserved for cases of severe toxicity. Further research on the effect of higher blood flow rates may lead to a more effective use of CVVHD in case of caffeine intoxication.

## Figures and Tables

**Table 1:** Laboratory results (No indication for follow-up or previously normalized).

	Day 1	Day 2	Reference Values
Hb (mmol/L)	12.5	10	8.5–11
Leucocytes (X 10^9^/L)	34.4	27.9	4.0–10.0
Sodium (mmol/L)	142	140	135–147
Potassium (mmol/L)	2.6	3.9	3.5–5.0
Phosphate (mmol/L	0.53	0.66	0.70–1.50
Magnesium (mmol/L)	0.68	0.87	0.70–1.00
Glucose (mmol/L)	11.6	6.8	4.0–7.8
Creatinine (umol/L)	137	75	59–104
AST (U/L)	42	-	<35
ALT (U/L)	92	-	<45
Creatine kinase (U/L)	483	-	<171
pH	7.55	7.51	7.35–7.45
Bicarbonate (mmol/L)	21.1	22.4	22.0–29.0
Lactate (mmol/L)	5.0	1.7	0.5–1.7
CK-MB (ug/L)	16	-	<7.6
Hs-trop T (ug/L)	0.066	-	<0.014

Hb: haemoglobin, AST: aspartate transaminase, ALT: alanine transaminase, CK-MB: creatine kinase-myocardial band, HS-trop T: high-sensitive troponin T.

**Table 2:** Pharmacokinetic parameters of caffeine blood concentration model.

Parameter	Population Value	Individual Value
Unbound fraction	0.64	0.64
Distribution volume	0.61 L/kg	0.86 (non-Bayesian) L/kg
Elimination constant	0.136 h^−1^	0.0462 h^−1^
Bioavailability	1	1
Absorption constant	19.8 h^−1^	19.8 h^−1^

## Data Availability

Not applicable.
